# The Effectiveness of the Use of Silver Fluoride and Teledentistry to Manage and Prevent Childhood Caries Among Aboriginal Children in Remote Communities: Protocol for a Cluster Randomized Controlled Trial

**DOI:** 10.2196/72227

**Published:** 2025-10-07

**Authors:** Peter Arrow, Susan Piggott, Lorraine Anderson, Dawn Bessarab, Lisa Jamieson, David Atkinson, Hien Ngo, Sanjeewa Kularatna, Utsana Tonmukayakul, Soniya Nanda, Jilen Patel, Lorraine Powell, Mohamed Estai

**Affiliations:** 1 Dental Health Services Government of Western Australia Department of Health Perth Australia; 2 University of Western Australia Perth Australia; 3 Dental School University of Adelaide Adelaide Australia; 4 Kimberley Aboriginal Medical Services Broome Australia; 5 Faculty of Medicine and Dentistry University of Western Australia Perth Australia; 6 National University Singapore Singapore Singapore; 7 Deakin University Burwood Australia; 8 Government of Western Australia Department of Health East Perth Australia; 9 Independent Consultant Perth Australia; 10 WA Country Health Service Government of Western Australia Department of Health East Perth Australia

**Keywords:** caries prevention, early childhood caries, Indigenous oral health, minimally invasive dentistry, silver fluoride

## Abstract

**Background:**

Australian Aboriginal children experience dental decay at more than twice the rate of non-Aboriginal children. The Select Committee into the Provision of and Access to Dental Services in Australia noted that the rate of potentially preventable hospitalizations was the highest among children aged between 5 and 9 years and was higher among Indigenous Australians and those living in remote locations. The application of a silver fluoride (AgF) solution to decayed surfaces has been shown to be effective in stopping the decay process and reducing the occurrence of new decay but has been tested to a limited extent in the Australian context.

**Objective:**

This study aims to evaluate the feasibility of using the skills of an Aboriginal health practitioner to undertake the application of AgF to carious primary molars to arrest the caries progression and prevent the occurrence of new caries among young Aboriginal children in remote communities.

**Methods:**

This study is a cluster-randomized controlled trial with communities randomized and stratified based on caries level and water fluoridation status. The trial will recruit 640 children (aged between 6 months and 7 years) from 30 communities. Informed consent will be obtained. At baseline, each child in the intervention group will be examined by a calibrated examiner and subsequently by an oral health practitioner who will prescribe to an Aboriginal health practitioner the teeth to be treated with AgF. A formulation with 38% AgF will be applied for 1 minute (0.004 mL per tooth). The control group will be provided with standard minimally invasive care. Participants will be followed annually for 2 years to assess caries arrest and prevention by blinded calibrated examiners. Child oral health–related quality of life and dental anxiety will be elicited through validated questionnaires. Tests of proportions will be used to evaluate the proportion of lesions arrested and the proportion of surfaces at risk that decayed over the follow-up. Multiple logistic regression with appropriate control for clustering of teeth and communities will be used to evaluate caries arrest, controlling for potential confounding factors.

**Results:**

Community engagement has commenced, and data collection protocols have been prepared. Staff specific to the study (eg, Aboriginal health practitioners or workers) are in the process of recruitment. Participant recruitment will commence in March 2026 and conclude in December 2026. Study outcomes will be reported at 12- and 24-month follow-ups.

**Conclusions:**

This study will test the effectiveness and feasibility of a non-oral health professional applying AgF to achieve caries arrest and prevention and validate clinical findings against digital imagery acquired on site. This pragmatic study will inform the development of suitable and accessible models of care for dental service provision in rural and remote communities in Australia.

**Trial Registration:**

Australian New Zealand Clinical Trials Registry (ANZCTR) ACTRN12624000457549p; https://www.anzctr.org.au/Trial/Registration/TrialReview.aspx?id=387518&isReview=true

**International Registered Report Identifier (IRRID):**

PRR1-10.2196/72227

## Introduction

### Background and Rationale

The interim report of the Select Committee into the Provision of and Access to Dental Services in Australia noted that the rate of potentially preventable hospitalizations was the highest among children aged between 5 and 9 years and was higher among Indigenous Australians and those living in remote locations [1]. The higher tooth decay experience of Australian Aboriginal children, in which the majority of the disease remains untreated [2], is believed, partly, to be due to unfavorable visiting patterns to dental care providers, associated with poor accessibility, cost, and the lack of cultural awareness by service providers [3,4]. Australian Aboriginal children experience dental decay at more than twice the rate of non-Aboriginal children. The current models of service delivery are failing to meet the needs, and most decayed teeth remain untreated. The management of dental decay in young children is challenging, and it is commonly undertaken under hospital-based dental general anesthesia.

A recently completed clinical randomized controlled trial (RCT) successfully managed dental treatment of children recommended for dental general anesthesia using minimally invasive dental treatment approaches in a primary care setting (atraumatic restorative treatment [ART] and Hall technique [HT]) [5]. An RCT in remote Aboriginal communities in Western Australia demonstrated improved access to culturally appropriate care with a greater level of dental care provided, with a reduction in the occurrence of new dental decay among preschool-aged children using the ART and HT approach [6]. Although the ART and HT approach improved access to care, it still required an oral health professional team to visit the remote communities and deliver the services. This limits access to services only when the clinical team is in the community and increases the costs of service delivery. The application of a silver fluoride (AgF) solution to decayed surfaces has been shown to be effective in stopping the decay process and reducing the occurrence of new decay but has been tested to a limited extent in the Australian context. The application of the AgF solution is simple, quick, painless, and noninvasive and could be undertaken by an appropriately trained non-oral health professional. A major disadvantage of the use of AgF is the staining of the treated lesion with a dark brown or black color. However, the additional application of potassium iodide (KI) immediately after the application of AgF has been promoted to reduce the staining effects [7,8].

The application of teledentistry and telediagnosis may also offer opportunities to improve service delivery in remote settings by using telecommunication technology to provide oral health care over a distance and diagnoses based on information collected via smartphone technology [9]. Systematic reviews have suggested that diagnostic accuracy in using photographic images for oral diagnosis is comparable to that of traditional clinical examination, and its use may offer opportunities to improve equity and efficiency in service provision in low-income and challenging access settings [10,11].

### Objectives

The principal aim of the proposed study is to evaluate the feasibility of using the skills of an appropriately trained Aboriginal health practitioner or worker to undertake the application of AgF to carious primary molars of young Aboriginal children in remote communities and evaluate the effectiveness and acceptability of AgF, using a 2-arm cluster RCT. It will also validate the use of teledentistry to diagnose and prescribe treatment remotely. The outcomes will be compared with those of the alternative care model using the recently tested ART and HT approach, delivered by an oral health professional team, for clinical outcomes, cost-effectiveness, cost utility, child oral health–related quality of life (COHRQoL), and child dental anxiety. The objectives of the project are as follows:

The first objective is to measure the number of teeth and tooth surfaces with arrested carious lesions and restored at baseline and follow-up. The hypothesis is that *the number of arrested carious lesions will be greater in the intervention group at follow-up.*

The second objective is to measure the number of teeth and tooth surfaces with dental decay and new decay and the number of teeth with dental infections among study participants at baseline and follow-up. The hypothesis is that *the number of children with newly active decay in their teeth and tooth surfaces and new dental infections in the intervention group will not be greater than the specified difference (risk difference) deemed to be a noninferior outcome.*

The third objective is to measure the extent of discoloration in teeth treated with AgF, with or without KI. The hypothesis is that *the extent of discoloration will be less in the intervention group treated with AgF and KI.*

The fourth objective is to measure the change in COHRQoL and dental anxiety using validated measures. The hypothesis is that *the extent and number of children with improved COHRQoL and dental anxiety in the intervention group will not be any less than the specified difference (risk difference) deemed to be noninferior.*

The fifth objective is to evaluate the cost-effectiveness and cost utility of the interventions. The hypothesis is that *the intervention will be less costly with a noninferior outcome.*

The sixth objective is to evaluate the acceptability of the interventions between the 2 groups of participants through focus group interviews using the “yarning” approach [12].

The seventh objective is to evaluate the reliability and validity of diagnosis and treatment prescription using teledentistry. The hypothesis is that the *sensitivity and specificity will be within the published limits for visual assessment of caries*, that is, 0.86 (95% CI 0.80-0.90) and 0.77 (95% CI 0.72-0.82), respectively.

## Methods

### Trial Design

This study will use a robust, pragmatic, 2-arm parallel group cluster randomized, active-controlled, noninferiority trial design. This study will be reported according to the CONSORT (Consolidated Standards of Reporting Trials) guidelines on reporting of noninferiority trials [13]. Study communities will be randomized into test or control groups by a central study coordinator through a computer-generated block randomization procedure, stratified by expected decay experience and exposure to community water fluoridation levels based on findings from our earlier work in the Kimberley region [6]. Both arms of the study will be provided with dental care; the control arm will be treated using the ART and HT approach, and the test arm will be treated using AgF. The test group will be further randomized into 2 groups via computer-generated allocation in concealed envelopes at study sites to receive 1 of the 2 AgF formulations. One formulation will have 38% AgF and KI as a staining reducing agent (Riva Star Aqua; SDI Limited); the other formulation will have 40% AgF with stannous fluoride as a reducing agent (Creighton Dental AgF; Whiteley Corporation). The Riva Star Aqua group will be further randomized to receive KI or no KI to evaluate KI’s effect on reducing the staining associated with AgF and evaluate its impact on caries arrestment. Moreover, we will evaluate the COHRQoL [14] and child dental anxiety and undertake an economic evaluation of the interventions. Focus group interviews, using the yarning approach, will be conducted to better understand the impact of the dental treatments [12]. We will also test the effectiveness of teledentistry in caries diagnosis and treatment prescription. Participants will have digital images taken of their dentition with a smartphone using standardized procedures [15]. The images will be stored and sent securely to a calibrated assessor at a remote site. The assessor, blinded to group allocation, will evaluate the images and diagnose and prescribe treatment.

### Study Setting

The interventions will be undertaken in remote community settings using mobile portable equipment in the Kimberley region of Western Australia. Because of the small population in the participating communities and to ensure confidentiality of participant information, the list of study sites will not be made available. The communities selected for inclusion in the study generally do not have ready access to dental care and may have to travel long distances by road to obtain care because care providers are not usually available in the community.

### Eligibility Criteria

Communities that participated in an earlier study will be invited to participate via community consultations with the community chair and community council [6]. Children aged between 6 months and 7 years, with or without dental caries, will be eligible. Children with complex medical conditions or developmental syndromes requiring specialist care or those with a need for urgent dental care will be excluded (those requiring urgent care will be referred to their local service provider).

### Ethical Considerations

This study received ethics approval from the Western Australia Aboriginal Health Ethics Committee (1348) and the University of Western Australia Human Research Ethics Office (2024/ET000760). A 2-stage consent process will be used. First, informed community consent will be obtained from the community to participate in the study. After community consent, individual signed informed consent will be obtained. The research team’s Aboriginal research assistant will assist parents and guardians by explaining the study to those whose first language is not English. Each participating parent and carer will be provided with a local community store voucher worth Aus $50 (US $32) as compensation for their time. Confidentiality of participant information will be protected by storing information on password-protected computers that are locked in offices accessible only to research personnel. Analysis will be undertaken on deidentified data.

### Interventions

#### Explanation for the Choice of Comparators

The primary comparison group is composed of the children who were provided with the minimally invasive treatment approaches, encompassing the ART [16] and the HT stainless steel crowns [17]. The ART and HT approach has been tested in remote communities and has been found to be an effective and feasible approach to delivering dental care in remote communities [6,18]. The secondary comparison is that of teeth treated with AgF with or without KI to evaluate the stain-reducing potential of KI.

#### Intervention Description

Children allocated to the test group will be provided with AgF, either Riva Star Aqua (38% AgF and KI) or Creighton Dental AgF (40% AgF and stannous fluoride). Only the primary molars will be included. The AgF will be applied by an Aboriginal health practitioner or worker who has been trained in the application of fluoride agents. The agents will be applied once only, after baseline assessment, and repeated at the 12- and 24-month follow-ups. All other treatments required beyond AgF application will be managed by the oral health practitioner team. Interventions for Riva Star Aqua and the Creighton AgF are outlined in Textbox 1.

**Interventions for Riva Star Aqua and the Creighton silver fluoride (AgF).**

**For Riva Star Aqua**

1. Remove food debris and plaque by brushing with a toothbrush (no toothpaste).
2. Isolate teeth to be treated with cotton rolls and gauze and apply petroleum jelly to the gingiva and lips.
3. Dry the affected tooth surfaces and at-risk tooth surfaces to be treated with cotton pledgets.
4. Dispense 0.05 mL AgF; bend the microbrush applicator and dip the tip of the applicator into the agent.
5. Apply the agent with the microbrush onto the decayed surface and rub gently, maintaining contact with the tooth for 60 seconds; apply the agent also to at-risk, non-decayed surfaces for 60 seconds and remove excess from all surfaces with cotton pledgets.
6. For tooth surfaces assigned to receive potassium iodide (KI), dispense 0.1 mL KI; using a new microbrush, dip the brush tip into the agent and apply to the decayed surface until precipitate forms. Then, reapply the agent until no precipitate forms, with 5- to 10-second intervals between reapplications.
7. Place Orabase adhesive (Colgate Oral Pharmaceuticals, Inc) onto AgF-treated decayed surfaces, with or without KI.
8. Advise participant to refrain from eating or drinking for 30 minutes after treatment.

**For Creighton Dental AgF**

1. Remove food debris and plaque by brushing with a toothbrush (no toothpaste).
2. Isolate teeth to be treated with cotton rolls and gauze and apply petroleum jelly to the gingiva and lips.
3. Dry affected tooth surfaces and at-risk tooth surfaces to be treated with cotton pledgets.
4. Dispense 0.05 mL AgF; bend the microbrush applicator and dip the tip of the applicator into the agent.
5. Apply the agent with the microbrush onto the decayed surface and rub gently, maintaining contact with the tooth for 60 seconds. Apply the agent also to at-risk, nondecayed surfaces for 60 seconds and remove excess from all surfaces with cotton pledgets.
6. Dispense 0.05 mL stannous fluoride. Using a new microbrush, dip the microbrush tip into the stannous fluoride solution and apply it to the decay-treated tooth surfaces.
7. Place Orabase adhesive onto treated decayed surfaces.
8. Advise participant to refrain from eating or drinking for 30 minutes after treatment.

Children allocated to the control group will be provided treatment by the oral health practitioner team (dental therapist and dental clinic assistant). Teeth to be restored using the ART approach will have the decayed tooth surfaces prepared using hand instruments only for caries removal without local anesthesia and teeth restored with a glass ionomer cement. Teeth restored using the HT will have the tooth surface cleaned, but caries will not be removed. The tooth will then be restored with a stainless-steel crown, cemented with a glass ionomer cement. The treating clinician will determine the type of restoration to provide for each control participant.

All the children will be reviewed at 12 and 24 months, and AgF treatments will be reapplied.

#### Criteria for Discontinuing or Modifying Allocated Interventions

Participants allocated to the test arm (AgF treatment) who request restorative care as per the control arm (ART and HT treatment) will be provided with the requested care. Similarly, control participants who request the test intervention will be provided with the test intervention. The reasons for the request will be noted. All participants will be analyzed on an intention-to-treat and per-protocol basis.

#### Strategies to Improve Adherence to Interventions

All participants will be provided with information and guidance on oral health care procedures. No specific actions are required from the participants beyond oral hygiene measures to maintain oral health.

#### Relevant Concomitant Care Permitted or Prohibited During the Trial

All participants will be able to access and receive regular dental care from their usual dental care provider. Information regarding treatment received during the follow-up interval will be sought from participants at each follow-up.

#### Provisions for Posttrial Care

All participants will be provided with contact details of the researchers to report any adverse events that may arise after the intervention.

### Outcomes

The primary outcome of interest is the number of treated decayed teeth at baseline that became arrested at each follow-up period and the number of sound surfaces treated at baseline that became carious at follow-ups, measured by the count of decayed, missing, and filled tooth surfaces. Secondary outcomes will be changes in COHRQoL and child dental anxiety (Table 1).

**Table 1:** Standard Protocol Items: Recommendations for Interventional Trials (SPIRIT) participant timeline.

SPIRIT participant timeline		Enrollment	Allocation	After allocation			Closeout
		–T1^a^		Baseline	12 mo	24 mo	
**Enrollment**							
	Community consent	✓					
	Individual informed consent	✓					
	List other procedures	✓					
	Allocation		✓				
**Interventions**							
	AgF^b^			✓	✓	✓	
	AgF and KI^c^			✓	✓	✓	
**Assessments**							
	List baseline variables			✓^d,e,f^	✓^d,e,f^	✓^d,e,f^	
	List outcome variables	✓	✓	✓^d,e,f^	✓^d,e,f^	✓^d,e,f^	
	List other data variables				✓^g^		

^a^T1: enrollment.

^b^AgF: silver fluoride.

^c^KI: potassium iodide.

^d^DMFS: decayed, missing, and filled tooth surfaces.

^e^COHRQoL: child oral health–related quality of life.

^f^Dental anxiety.

^g^Focus group.

### Sample Size

We used the *power* command within Stata (version 15; StataCorp) and data on decay incidence, restorative outcomes, and COHRQoL impacts from the recently completed Kimberley study [6] to estimate the noninferiority sample size. Approximately 61% of the ART restorations were classified as successful (lesion arrestment), and 81% were estimated to be arrested with AgF treatment from published studies [19]; community intracluster correlation was 0.09. Using these parameters at 80% power and an α of .05 and with 30 clusters available (n=15, 50% clusters per treatment arm), the estimated sample size required was 180 in each arm. Allowing for a 25% loss to follow-up each year, the estimated sample size is 320 per arm or 22 children per cluster (rounded up). The sample size will also have 84% power to undertake subgroup analyses to detect a 20% difference in caries arrestment between carious lesions treated with AgF and KI or without KI. A sample size of 40 for the focus group interviews is expected to be sufficient to allow for the elicitation of parent perceptions about the care provided based on our earlier work [20,21].

### Recruitment

Strategies used in the recently (successfully) completed study in the Kimberley region will be adopted, with further community engagement undertaken to refine and develop the research protocol with the community leaders. Participant recruitment will commence after obtaining community consent to participate. Participant recruitment and retention strategies will include (but not be limited to) personal contacts by the project coordinator and the Aboriginal research assistant and engagement via the media (radio and television); website; local agencies, such as the community offices, the local school or schools, child care centers, community health and nursing centers; posters and flyers at local community stores, and word of mouth via the community and the AAC.

Parents of eligible children expressing an interest in participating will be provided with an information sheet, and the study will be explained by the Aboriginal research assistant. Upon receipt of signed consent, each parent will complete a questionnaire (the Aboriginal research assistant will assist the parent to complete the questionnaire). A calibrated examiner will then undertake the baseline clinical examination and take digital images of the dentition using a developed protocol.

**Figure 1:**
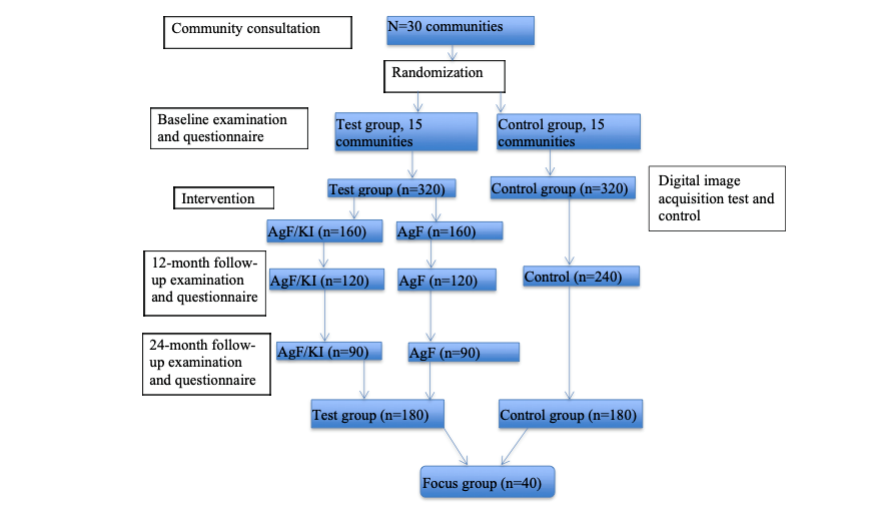
Participant flowchart. AgF: silver fluoride; KI: potassium iodide.

After the baseline examination, each child will be offered treatment as per the protocol for the group allocation. Efforts will be taken to provide comprehensive care, including extractions. From experience, we found that concurrent treatment of the child after the baseline examination offered the best option for care and reduced the likelihood of nonattendance at subsequent appointments. In addition, where extra capacity to deliver dental treatment is available, we will provide dental care to children not enrolled in the study as a community service. The CONSORT participant flowchart is shown in Figure 1.

### Assignment of Interventions: Allocation

#### Sequence Generation

Communities will be allocated to intervention or control groups through computer-generated block randomization, stratified based on the estimated caries prevalence and community water fluoridation levels within communities. Communities will be assigned to the intervention or the control group after receiving community consent. Individual children in the intervention group will be further allocated into AgF treatment or AgF and KI treatment via sealed envelopes containing computer-generated block randomization. The envelopes will be opened by the project coordinator at the community study site, and they will assign each child at enrollment.

#### Concealment Mechanism

The clinical team and the treating clinicians will be aware of the group assignment. Allocation assignment of KI after AgF application will be stored in opaque, sealed envelopes, which will be opened only at the time of treatment by the project coordinator.

#### Implementation

The allocation will be generated by the project lead off-site, and communities will be allocated into the intervention or control groups. Individual randomization will also be generated off-site and placed in sealed envelopes, which will be opened on-site by the project coordinator and assigned to AgF or AgF and KI treatment groups upon enrollment in the project.

### Assignment of Interventions: Blinding (Who Will Be Blinded?)

The nature of the intervention is such that participants, treating clinicians, and assessors will not be blinded to group allocation. The project analyst will be blinded to group allocation.

### Data Collection and Management

#### Plans for Assessment and Collection of Outcomes

Treated teeth with intact, sound fillings will be assumed to have arrested decay. The changes will be evaluated through a count of decayed, missing, filled, and arrested decayed tooth surfaces at baseline and 12- and 24-month follow-ups using the International Caries Detection and Assessment System criteria [22]. The assessment will be undertaken by calibrated examiners. Color changes of AgF-treated or AgF- and KI-treated lesions will be evaluated through the classification of the treated lesion into 4 categories according to Pantone color plates. Data will be collected through direct data entry using the REDCap (Research Electronic Data Capture; Vanderbilt University) implementation on iPads (Apple, Inc). The Aboriginal research assistant will assist parents, carers, and children whose primary language is not English in completing the questionnaire.

Parents of the study participants will be invited to take part in focus group interviews at the 12-month follow-up. Consenting parents will be interviewed by the Aboriginal research assistant using a semistructured interview schedule, and the interviews will be digitally recorded. The structured open-ended questions will elicit responses from parents on the care received by their child and follow a yarning approach [12]. The questions will be based on the following:

1. What were some of the positive aspects of dental care experienced by your child?
2. What were some of the negative aspects of the dental care received by your child?
3. Can you give some examples of what you think could have been done and implemented better during your child’s treatment?
4. Can you name some aspects of the setting, location, and process that you think could have been improved?
5. Can you identify any changes to your oral health knowledge since the research began?

We will also take digital images of the teeth of selected children who provide consent to have the images of their teeth recorded using a smartphone and a standardized protocol [15]. The images will be sent via email, using a secure protocol used within the Western Australia Health Department, to a remote site where an independent, calibrated assessor will assign scores to the images using the same criteria as the field assessment.

#### Plans to Promote Participant Retention and Complete Follow-Up

Participant retention will be implemented following the methods adopted in our previous project, which included communication via the project’s Facebook account and regular information dissemination to the participating communities through the study community reference group. Direct contact with study participants will also be maintained through email contacts.

#### Data Management

Data will be collected through the REDCap implementation on an iPad. Data entries will have range checks and data validation. All data entries will be performed via a password-protected iPad, which will be stored in locked cabinets situated within locked offices, accessible only to the research personnel.

### Statistical Methods

#### Statistical Analyses for Primary and Secondary Outcomes

Analyses will be on an intention-to-treat and per-protocol basis. Objectives 1 to 3 will be tested using a test of proportions and logistic regression and negative binomial regression for overdispersed count data, accounting for clustering within individuals and communities. Subgroup objective 4 will be tested using paired tests for pre- and postintervention changes in COHRQol and dental anxiety as well as unpaired tests for between-group differences at baseline and follow-up; multivariable analyses will use generalized estimating equation linear regression to control for teeth and community clustering. Objective 5, economic analyses, will be tested as outlined in the subsequent section. All analyses will be undertaken by analysts who are blinded to group allocation status, and take into account clustering within communities and baseline factors and any baseline intergroup differences. Multiple imputations of missing data will be undertaken to evaluate its impact on the primary and secondary outcomes.

Objective 6 will be evaluated through qualitative analyses of the focus group interviews, with all interviews digitally recorded and independently transcribed. Transcripts will be read, and coding will be undertaken by analysts who are blinded to group allocation status. A thematic analytical approach will be adopted to develop themes and explore the contextual differences among the perceptions, experiences, and beliefs without a strict requirement for a theoretical focus [23,24]. Transcripts will be read multiple times to establish familiarity with the dataset. Two independent reviewers, one of whom will be an Indigenous researcher, will develop a coding scheme of the transcripts and also develop emerging themes. Where disagreement in coding is identified, the differences will be resolved through discussion and consensus. Our approach will broadly follow a reflexive thematic analysis (based on the study by Braun and Clarke [24]), drawing on subjective interpretations of both an Indigenous and a non-Indigenous researcher. The emergent themes will be explored and further refined. Data management will be facilitated using NVivo (version 12; Lumivero). Objective 7 will be evaluated by calculating sensitivity, specificity, predictive values, and receiver operating characteristics, validated against on-site clinical assessment.

#### Economic Analysis

Costs and outcomes of the interventions will be quantified and analyzed. The costs of the intervention and subsequent dental treatment costs within the trial period will be assessed from the health provider’s perspective. The intervention cost will be recorded using a World Health Organization–developed program costing template called CostIt, using a bottom-up approach. Unit costs of the intervention, for example, salary and AgF, will be based on the budget record of the study. The costs of dental treatments required will be determined using the treatment item codes extracted from the participant’s dental records and unit costs based on the Child Dental Benefit Schedule. Cost-effectiveness and cost-utility analysis will be undertaken. The incremental cost-effectiveness ratio or the changes in net costs and health outcomes resulting from the intervention compared with the control will be calculated. The incremental cost-effectiveness ratio can be presented as follows:

ICER = (Ci – Cc) / (Ei – Ec)

where Ci and Ei are the net costs and effectiveness of the intervention and Cc and Ec are those of the control. In the cost-effectiveness analysis, the E component is a clinical outcome. For the cost-utility analysis, the outcome is the number of years weighted by the quality-of-life index derived from ECOHIS-4D [25]. A standard World Bank and World Health Organization Choosing Interventions that are Cost-Effective (WHO-CHOICE) discount rate of 3% will be applied to both costs and outcomes that occur after the first year [26]. Uncertainty analysis will be performed.

#### Plans to Give Access to the Full Protocol, Participant-Level Data, and Statistical Code

Clinical information of the child study participants will be shared with their parents and carers at the examination presentation. The results of this study will be shared widely through publication of the findings in scientific journals and conference presentations.

### Oversight and Monitoring

#### Composition of the Coordinating Center and Trial Steering Committee

All investigators will comprise the trial steering committee and will contribute to overall monitoring of the study and guide its conduct. The committee will meet quarterly, and the lead investigator will report on the progress of the study to date.

#### Community and Consumer Engagement

At the completion of our earlier work in the Kimberley region, we provided community feedback to the communities that participated in our study through the community council and the local Aboriginal community-controlled health organizations [6]. Because of the community feedback, a proposal was formulated to overcome the sole reliance on visiting dental service providers for primary dental care by using the skills of an Aboriginal health practitioner or worker. The proposal was endorsed by the local Aboriginal community–controlled health organizations, and the resulting project was developed. An Aboriginal advisory committee will also be formed and provide guidance on the research process and ensure cultural safety during the trial. We have also engaged with a community consultant (LP) who read the study proposal and provided input for the study.

#### Composition of the Data Monitoring Committee, Its Role, and Reporting Structure

An independent data and safety monitoring committee will also be convened, comprising 3 independent senior clinicians, who will review clinical outcomes and, in particular, any reported adverse events to protect and ensure participant welfare.

#### Adverse Event Reporting and Harms

Participants will be assessed immediately after the treatment for any adverse events, and parents and carers of the child study participant will be provided with contact details of the research team for reporting any adverse events that occur after the treatment. Reporting of any adverse events will be closely monitored and reported to the data and safety monitoring committee on a quarterly basis.

#### Frequency and Plans for Auditing Trial Conduct

The lead investigator will provide quarterly reports to the steering committee, which will meet quarterly and appraise the trial conduct.

#### Plans for Communicating Important Protocol Amendments to Relevant Parties (eg, Trial Participants and Ethics Committees)

Any amendments and alterations to the study protocol will be reported to the appropriate institutional ethics committee.

#### Dissemination Plans

The study findings will be reported back to participating communities in a grouped form, and no individual community or individual will be identifiable. The findings will be further reported in appropriate scientific forums, including conferences and journals. A report will also be prepared and provided to the supporting organizations.

## Results

Community engagement has commenced, and we have approached communities that participated in our earlier project for participation in the proposed project. The trial has been registered on the Australian New Zealand Clinical Trials Registry on April 15, 2024 (ACTRN12624000457549p). Recruitment of participants is planned to commence on March 1, 2026, and is expected to be concluded by December 31, 2026.

Data collection tools via REDCap have been prepared. Calibration of clinical examiners is in progress. The study outcomes with respect to oral health will be published after the 12- and 24-month follow-ups have been completed. The qualitative findings will be reported after the focus group discussions have been completed.

## Discussion

### Anticipated Findings

Access to timely dental care is a challenging issue confronting many people in rural and remote communities in Australia but is particularly acute for the Indigenous population. The remote communities tend to have smaller population sizes, and there are usually no dental services available within the community; the residents of the community will usually have to travel long distances to obtain care or wait for itinerant visits by public dental services or volunteer-based service providers. This then leads either to a delay in getting care or, worse, trying to access care only when in pain, which can sometimes lead to the required care only being able to be provided under general anesthesia. By using the skills of an Aboriginal health worker or practitioner, who would normally reside within a community, to deliver primary dental care, it is expected that the intervention will promote better access to timely dental care, thus mitigating the factors outlined earlier.

The use of AgF to stop caries progression has been tested worldwide and has been shown to be effective in arresting caries. The procedure is relatively simple and painless. The simple and painless application lends itself to use among populations where standard care options for the management of dental caries may not be feasible or practical and to test its potential application by an appropriately trained non-oral health practitioner. This study will test the application of AgF by an Aboriginal health worker or practitioner.

This trial will test the feasibility and effectiveness of applying AgF to carious primary molars of young Indigenous children in remote communities. The rate of arrestment will be compared with the rate of arrestment among children in control communities provided with the minimally invasive care using the ART and the HT. We will also test the feasibility of using digital images acquired via a smartphone to validate on-site clinical assessments undertaken by calibrated examiners. The findings have the potential to disrupt a model of care that relies principally on care delivered by an oral health professional team using local anesthesia and the dental drill. Validation of remote clinical assessment for dental conditions will enable the use of teledentistry technology to further improve access to dental care in remote communities by reducing the reliance on care delivered by visiting dental teams.

### Conclusions

This trial is set within remote communities in Western Australia and among young children; therefore, the findings may not necessarily be directly applicable to other settings and age groups. However, residents of all ages in remote communities in Australia, in general, confront similar difficulties and challenges in accessing timely dental care, and this study’s findings may inform the consideration of policies and practices for service delivery in such settings.

## Appendix

Multimedia Appendix 1 Peer review report from the 2023 Partnership Projects PRC1 (National Health and Medical Research Council, Australian Government).

## Abbreviations

AgF: silver fluoride
ART: atraumatic restorative treatment
COHRQoL: child oral health–related quality of life
CONSORT: Consolidated Standards of Reporting Trials
HT: Hall technique
KI: potassium iodide
RCT: randomized controlled trial
REDCap: Research Electronic Data Capture
WHO-CHOICE: World Health Organization Choosing Interventions that are Cost-Effective

## Data Availability

The datasets generated or analyzed during this study are not publicly available due to confidentiality, but are available from the corresponding author on reasonable request.
