# Supplementary material for: The Effectiveness of the Use of Silver Fluoride and Teledentistry to Manage and Prevent Childhood Caries Among Aboriginal Children in Remote Communities: Protocol for a Cluster Randomized Controlled Trial
Source: JMIR Res Protoc. 2025 Oct 7;14:e72227. doi: 10.2196/72227 (PMC12541258; doi:10.2196/72227)
Supplement: Multimedia Appendix 1 [file resprot_v14i1e72227_app1.pdf]

Australian Government
National Health and Medical Research Council

NHMRC

Application Assessment Summary

2023 Partnership Projects PRC1

Application ID: 2025089
Chief Investigator A: Doctor Peter Arrow
Administering Institution: University of Western Australia

Table 1: Summary of the assessment of your application against the **2023 Partnership Projects PRC1** Grant Assessment Criteria.

Criteria		
1	1. Track records of the Chief Investigators, Partner Organisations and Partner Investigators, relative to opportunity (25%)	5.800
2	2. Scientific quality of the proposal and methodology (25%)	5.400
3	3. Relevance and likelihood to influence health policy and practice (25%)	5.900
4	4. Strength of the partnership (25%)	5.900
	**Overall Score**	**5.750**

Table 2: Summary of assessments by the **2023 Partnership Projects PRC1** Peer Review Panel (PRP).

Table 2 provides a summary of Partnership Project application scores for this peer review cycle following discussion by the Peer Review Panel. It does not include applications deemed ‘Not For Further Consideration’ after initial assessment. The proportion of applications and mean scores for each criterion are provided for each overall score range.

Overall score range	% of apps in overall score range	Criterion 1 (mean)	Criterion 2 (mean)	Criterion 3 (mean)	Criterion 4 (mean)
6.501 - 7.000	Nil	Nil	Nil	Nil	Nil
5.501 - 6.500	11.54%	5.867 ± 0.047	5.567 ± 0.125	5.667 ± 0.170	6.067 ± 0.125
4.501 - 5.500	73.08%	5.383 ± 0.414	4.298 ± 0.454	4.544 ± 0.502	5.304 ± 0.328
3.501 - 4.500	15.38%	4.404 ± 0.581	3.664 ± 0.409	3.664 ± 0.290	4.501 ± 0.279
<3.501	Nil	Nil	Nil	Nil	Nil

Table 3: Peer Reviewer Comments
Qualitative feedback on your application is provided below in the form of Peer Reviewer comments. The feedback provided by peer reviewers is not the view of NHMRC. It is the expert opinion of the peer reviewers who assessed the application.

For more information on the steps NHMRC has taken to support quality peer review, visit:
https://www.nhmrc.gov.au/funding/peer-review/disclaimer

Peer Reviewer Comments:
**Assessor Role:** Spokesperson 1<br>**Question:** 1. Track records of the Chief Investigators, Partner Organisations and Partner Investigators, relative to opportunity (25%)<br><br>CIA has led 4 RCT's as a Dentist working in remote communities; Other CI's have relevant expertise in clinical care, Aboriginal health and in positions of authority able to influence clinical practice changes post research completion. IREC review positive. Great levels of co-design and experience over time of meaningful community engagement. Aboriginal CI's leading project activities with partner organisations well positioned for translation of findings that can have important and wide-spread impacts on clinical practice and community outcomes. Partners are government bodies and Aboriginal community organisations well positioned to integrate outcomes.
**Assessor Role:** Spokesperson 1<br>**Question:** 2. Scientific quality of the proposal and methodology (25%)<br><br>A two-arm delayed intervention cluster RCT design proposed to investigate clinical outcomes, QoL, child dental anxiety and economic impacts. Also evaluating feasibility of AHP and acceptability of AgF treatment. Acceptability measured using 'yarning approach'. Insufficient details in data analysis, e.g., how will the Aboriginal lens be integrated for data analysis using the yarning approach, will it be conducted by Aboriginal investigators, how will themes be explored? Good to see sample adjustments for clustering. Economic analysis appropriate, with CI's with economic expertise to lead the evaluation. Feasibility of participant recruitment in the time frame not justified. Some concern about compliance of regime. But qualitative assessments may help to unpack this. Small sample size – quality of life measure may not be sufficient to undertake the quality of life analysis. It's novel by disrupting existing models of care. – inferiority margins not explicitly stated for qol. Some concerns about governance. Does require robust coordination given large number of stakeholders involved.
**Assessor Role:** Spokesperson 1<br>**Question:** 3. Relevance and likelihood to influence health policy and practice (25%)<br><br>Investigators and partners are well positioned in clinical care and community health services to implement changes to service delivery following results of this research trial. Some outline of implementation pathways are reported, however, they lack sufficient detail in the evidence translation strategy regarding barriers and enablers that may support translation int his context. However, investigator past history of translation into policy and practice demonstrate success nationally in the dental field. Great transferability.
**Assessor Role:** Spokesperson 1<br>**Question:** 4. Strength of the partnership (25%)<br><br>Strong partnership across government branches in health and dentistry and within local Aboriginal community organisations. This is demonstrated through investigator long-standing roles within these organisations and the level of cash and in-kind contributions offered by the participating organisations.
